# Mechanisms of pressure-mediated cell death and injury in *Escherichia coli*: from fundamentals to food applications

**DOI:** 10.3389/fmicb.2015.00599

**Published:** 2015-06-24

**Authors:** Michael Gänzle, Yang Liu

**Affiliations:** Department of Agricultural, Food and Nutritional Science, University of Alberta, Edmonton, AB, Canada

**Keywords:** *Escherichia coli*, EHEC, STEC, high hydrostatic pressure, food preservation

## Abstract

High hydrostatic pressure is commercially applied to extend the shelf life of foods, and to improve food safety. Current applications operate at ambient temperature and 600 MPa or less. However, bacteria that may resist this pressure level include the pathogens *Staphylococcus aureus* and strains of *Escherichia coli*, including shiga-toxin producing *E. coli*. The resistance of *E. coli* to pressure is variable between strains and highly dependent on the food matrix. The targeted design of processes for the safe elimination of *E. coli* thus necessitates deeper insights into mechanisms of interaction and matrix-strain interactions. Cellular targets of high pressure treatment in *E. coli* include the barrier properties of the outer membrane, the integrity of the cytoplasmic membrane as well as the activity of membrane-bound enzymes, and the integrity of ribosomes. The pressure-induced denaturation of membrane bound enzymes results in generation of reactive oxygen species and subsequent cell death caused by oxidative stress. Remarkably, pressure resistance at the single cell level relates to the disposition of misfolded proteins in inclusion bodies. While the pressure resistance *E. coli* can be manipulated by over-expression or deletion of (stress) proteins, the mechanisms of pressure resistance in wild type strains is multi-factorial and not fully understood. This review aims to provide an overview on mechanisms of pressure-mediated cell death in *E. coli*, and the use of this information for optimization of high pressure processing of foods.

## Introduction

Processing with high hydrostatic pressure in the range of 400–600 MPa has become a commercially viable unit operation in food production. The commercial application of pressure processing since the early 1990 ties was favored by an increasing body of research documenting beneficial effects on food quality and safety (Buckow et al., 2013; Balasubramaniam et al., 2015), the need to introduce alternative processing methods to maintain the safety of ready-to-eat foods (Gottlieb et al., 2006; Farber et al., 2011), and the increasing availability and suitability of commercial-scale high pressure equipment (Tonello, 2011). The commercial use of high pressure applications particularly includes its use as an alternative to thermal preservation (San Martín et al., 2002; Zhu et al., 2005; Tonello, 2011; Balasubramaniam et al., 2015). Pressure applications aiming to achieve food preservation are designed to obtain a bactericidal effect comparable to pasteurization but an improved retention of nutritional or sensory attributes when compared to thermally processed products (Tonello, 2011; Balasubramaniam et al., 2015; Georget et al., 2015).

Hydrostatic pressure in the range of 400–600 MPa inactivates food-borne viruses (Kingsley et al., 2002) and vegetative bacterial cells including many of the food-associated spoilage organisms and pathogens (Patterson et al., 1995; Garriga et al., 2004; Bièche et al., 2009; Jofré et al., 2010). Despite the current commercial use of pressure to eliminate bacteria in food, several concerns hamper the more widespread use of pressure in food preservation:

- Most bacterial endospores and few fungal ascospores resist pressure application at ambient temperature without loss of viability (Butz et al., 1996; Black et al., 2007). Pressure resistant ascospores and endospores are particularly relevant as spoilage organisms in fruit juices (Lee et al., 2006).
- The thermal elimination of microorganisms is typically predicted on the basis of *D*-and *z*-values that are derived from log-linear models. While this approach is error-prone, it is simple, robust, and of sufficient accuracy to allow the design of safe commercial processes. Despite numerous successful approaches to achieve a mathematical description of pressure-death-time data (Kilimann et al., 2006; Chen, 2007; Koseki and Yamamoto, 2007), these models do not exhibit sufficient simplicity, accuracy, or widespread acceptance to predict the bactericidal effect of pressure in commercial applications.
- The post pressure survival of target organisms is as relevant as the direct lethal effect of pressure treatment. Depending on the choice of food matrix, pH, and target organism, a post-treatment quarantine period allows for the elimination of surviving bacterial cells (Garcia-Graells et al., 1998; Jordan et al., 2001; Kilimann et al., 2005). However, post-pressure storage or incubation also supports resuscitation of injured cells that were undetectable after pressure treatment (Garriga et al., 2004; Koseki and Yamamoto, 2006).
- Bacterial resistance to pressure exhibits a large intra-species variability (Alpas et al., 1999; Benito et al., 1999; Liu et al., 2015) and particularly the species *Escherichia coli* and *Staphylococcus aureus* comprise strains that resist application of 600 MPa at ambient temperature with only a minimal reduction of cell counts (Alpas et al., 2000; Tassou et al., 2008). Pressure resistant mutant strains of *Listeria monocytogenes* and *E. coli* are readily isolated in the laboratory and wild type strains with a comparable and exceptional resistance to pressure occur in food (Hauben et al., 1997; Karatzas and Bennik, 2002; Liu et al., 2015). Validated strain cocktails for use in high pressure challenge studies have been described only for few bacterial species (Garcia-Hernandez et al., 2015).
- The bactericidal effect of pressure is highly dependent on the food matrix. The synergistic and antagonistic interactions of pressure and low pH, high temperature, and low water activity on bacterial inactivation are well understood (Garcia-Graells et al., 1998; Smelt, 1998; Molina-Gutierrez et al., 2002; Molina-Höppner et al., 2004). Effects of low-temperature treatments (Luscher et al., 2004), or additional interactions with the food matrix, however, are less well described and often require a case-by-case evaluation of the bactericidal effect of pressure in a given food matrix.

The further exploitation of pressure as preservation technology thus requires an improved understanding of pressure-mediated cell death and sublethal injury and the interaction of pressure with intrinsic or extrinsic factors prevailing in food. Recent reviews provide an excellent overview on the role of pH and water activity on the inactivation of vegetative bacterial cells and bacterial endospores (Georget et al., 2015). This communication aims to complement past reviews by providing an overview on the current knowledge of mechanisms of pressure-mediated cell death and injury and their relevance for food preservation, focusing on pathogenic *E. coli*. The physiology and genetics of this organisms are well understood, moreover, this species comprises strains that are of major public health concern (Croxen et al., 2013), as well as strains that exhibit exceptional resistance to pressure (Hauben et al., 1997; Vanlint et al., 2012; Liu et al., 2015).

## Pressure-mediated Elimination of *E. coli* in Food: An Overview

Numerous studies provide data on the inactivation of *E. coli* in food; Table 1 categorizes literature data by product type with reference to serotype and pathotype. Table 1 highlights the variability of the effects of pressure on *E. coli* in food, demonstrating that pressure effects are strain and matrix dependent. In each product category, some studies report a reduction of cell counts of less than 99% after treatment with 400–600 MPa at ambient temperature, while other studies report a reduction of cell counts exceeding 8 log(cfu/g) (Table 1). Likewise, treatment of the same strain in different food products at comparable conditions resulted in highly variable lethal effects (Table 1). Despite this substantial variability, three major trends can be derived from the data compiled in Table 1. First, studies employing strain cocktails or single strains selected for pressure resistance typically report lower process lethality when compared to studies employing single (outbreak) strains (Table 1). Second, the resistance of *E. coli* in meat and (fluid) milk is higher when compared to the resistance in low-pH fruit products. In meat and milk, treatments at 400–600 MPa at ambient temperature result in a reduction of cell counts by 5 to less than 1 log(cfu/g) while comparable treatments in some fruit juices reduced cell counts by more than 6 log(cfu/g). Third, treatment at elevated temperature (>40°C) greatly enhances process lethality and eliminates even pressure-resistant strains (Table 1). The combination of pressure treatment with elevated temperature and/or low pH, however, is not suitable for all food products and preservation of low-acid and temperature sensitive food thus required the identification of additional antimicrobial hurdles that act synergistically with pressure. Pressure sensitive targets in cells of *E. coli* and the possible exploitation of these targets for development of hurdle technologies are discussed in the subsequent sections.

**TABLE 1:** **Pressure-inactivation of different strains of *E. coli* in food**.

***Escherichia coli* serotype (number of strains) or strain number**	**P/T (MPa/°C)**	**Time (min)**	**Lethality^2^**	**Products (Reference)**
	**Meat and meat products**			
**O103:H5 (1)**	600/24–30	3.3	3.3	Sausage Omer et al. (2010)
**O157:H7 (4)**	600/28–37	1–5	>4.7	RTE meats Porto-Fett et al. (2010)
**O157:H7 (4) O157:NM**	600/34	3	4	RTE meats Gill and Ramaswamy (2008)
**O157:H7 (1)**	400/12	20 5 × 5 × 5	4.39 4.96	Ground beef Morales et al. (2008)
**O157:H7 (5)**	400/20 400/–5	10	3 1	Ground beef Black et al. (2010)
**O26, O121, O145 and O157 (4)**	600/25	3	2–6	Ground beef Liu et al. (2015)
**O26, O45, O103, O111, O121, O145, O157 (11)**	450/20	5	3.5–4.4	Ground beef Hsu et al. (2015)
AW1.7 and *LMM1030*	400/40	30	3–5	Ground poultry Liu et al. (2012)
PARC 449, 05-6544, 03-2832, 03-6430, and C0283 AW1.7, AS1.3, GM16.6, DM18.3, and MG1655	600/20	5	2 1.8	Ground beef Garcia-Hernandez et al. (2015)
**O157:H7** FDA5187	400/30	20	1	Ground beef Baccus-Taylor et al. (2015)
	**Milk and dairy products**			
**O59:H21 (1)** **O157:H7 (1)**	400/20	10	4.28 4.05	Cheese De Lamo-Castellví et al. (2006)
ATCC 11229	590/5	1 × 1 × 1	4	Milk Drake et al. (1997)
ATCC 43888	350/25	15	1	Skim milk Nakimbugwe et al. (2006)
**O157:H7 (2)**	350/50	5	>8	Milk Alpas and Bozoglu (2000)
ATCC 11303, ATCC 11775, MG1655, and ATCC 43888	550/20	15	2–6	Milk Garcia-Graells et al. (2000)
	**Fruit juices, vegetable, and fruit products**			
**O157:H7 (5)**	450/21	2	6	Strawberry puree Huang et al. (2013)
**O26, O45, O103, O111, O121, O145, O157 (11)**	450/20	5	>9	Strawberry puree Hsu et al. (2014)
**O157:H7 (2)**	350/50	5	>8 1–2	Orange juice Alpas and Bozoglu (2000) Orange juice
				
**O157:H7 (1)**	500/20	5	5	Tomato juice
			5	Apple juice Jordan et al. (2001)
MG1655,* LMM1010, LMM1030*	400/20 300/20	15 15	1 – >4 1 – >4	Orange juice Apple juice Garcia-Graells et al. (1998)
**O157:H7 (3)**	620/15	2	8.34 0.41	Grapefruit juice Grapefruit juice Apple juice Teo et al. (2001)
**O157:H7 (1)**	550/6	2	1.92	Apple juice Whitney et al. (2008)
**O157:H7 (6)**	550/6	2	1–4.4	Apple juice Whitney et al. (2007)
ATCC 25922	400/25	3	4.82	Cashew apple juice Lavinas et al. (2008)
**ATCC 11775**	300/20	5 5	4 1	Kiwi fruit juice Pineapple juice Buzrul et al. (2008) Pineapple juice Buzrul et al. (2008)
	400/25	10	5	Apple pieces
*LMM1010*	400/40	10	>7	Apple pieces
	400/40	10	5	Apple in 25% glucose Vercammen et al. (2012)
ATCC 25922, **O157:H7 (2)**	400/45 400/45	20 20	5.3 >7.7	Apple juice Ukuku et al. (2013)
**O104:H4**	400/42 300/50	10 10	3 3	Carrot juice (pH 5.1) Reineke et al. (2015)

^1^*VTEC are printed in bold; laboratory selected pressure resistant mutant strains are printed in italics.*

^2^*Lethality: Reduction of log(CFU/g) or log(CFU/mL).*

## Pressure Mediated Disruption of the Outer Membrane

**FIGURE 1:**
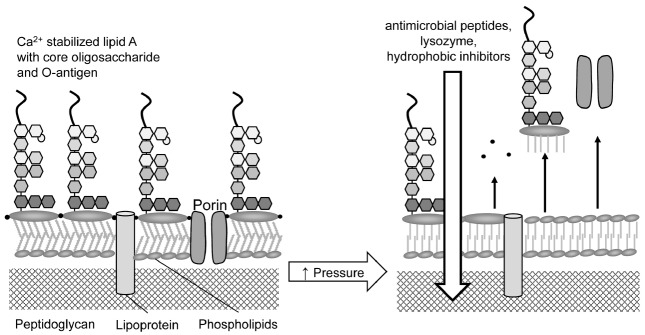
**Pressure effects on the outer membrane of *E. coli*.** The outer leaflet of the outer membrane is composed of a lipopolysaccharide layer which prevents penetration of large or hydrophobic molecules to the periplasm. Lipid A molecules are cross-linked by divalent cations. Porins provide channels for small hydrophilic compounds; lipoproteins that are anchored in the peptidoglycan stabilize the outer membrane. Pressure application disrupts the electrostatic interactions between divalent cations and negatively charged LPS molecules, resulting in dissociation of lipid A from the outer membrane and the integration of phospholipids in the outer leaflet. Outer membrane proteins also dissociate from the membrane (Ritz et al., 2000). This process permits entry of hydrophobic inhibitors (Kalchayanand et al., 1992; Hauben et al., 1996; Gänzle and Vogel, 2001). The uncommon porin NmpC may contribute to pressure resistance in *E. coli* AW1.7 (Ruan et al., 2011), and the porin OmpX is over-expressed in *E. coli* during grown at elevated pressure (Nakashima et al., 1995). Lipoproteins including OsmB and NlpI contribute to structural integrity of *E. coli*, and mediate pressure resistance (Charoenwong et al., 2011).

The barrier properties of the Gram-negative outer membrane mediate resistance against antimicrobial peptides including lysozyme, lactoferrin, and bacteriocins from lactic acid bacteria, and hydrophobic inhibitors including bile acids, which are ingredients of most selective media for *E. coli* or coliform bacteria (Vaara, 1992; Gänzle et al., 1999; Nikaido, 2003). The observation that pressure permeabilizes the outer membrane of Gram-negative bacteria was initially based on the synergistic activity of pressure and pediocin or nisin (Kalchayanand et al., 1992). Pressure application also sensitizes *E. coli* to lactoferrin and lysozyme, lactoferrin, and the lactoperoxidase system (Hauben et al., 1996; Garcia-Graells et al., 2000; Masschalck et al., 2001a,b). *In situ* determination of the permeabilization of the outer membrane suggested that the outer membrane is reversibly permeabilized concomitant with compression, followed by the irreversible loss of lipid A and outer membrane proteins (Figure 1; Ritz et al., 2000; Gänzle and Vogel, 2001). The outer membrane is stabilized by electrostatic interactions of Ca^2+^ and lipid A (Vaara, 1992); electrostatic interactions are pressure sensitive. Outer membrane porins are over-expressed during growth at elevated pressure (Nakashima et al., 1995) and the pressure-resistant strain *E. coli* AW1.7 is distinguished by most other strains of *E. coli* by expression of the porin NmpC (Ruan et al., 2011; Liu et al., 2012). The outer membrane lipoproteins NlpI, YbaY, and OsmE increase pressure resistance of *E. coli*, presumably through stabilization of the outer membrane (Charoenwong et al., 2011).

Pressure-mediated disruption of the outer membrane does not compromise the viability of *E. coli*, however, it may allow synergistic elimination of *E. coli* by combination of pressure with outer membrane-impermeant inhibitors. The synergistic activity of pressure with bacteriocins, lactoferrin, or the lactoperoxidase system was demonstrated in selected applications. Bacteriocin-producing cheese starter cultures acted synergistically with pressure to eliminate *E. coli* O157:H7 in raw milk cheese (Rodriguez et al., 2005), the differential inactivation when compared to non-bacteriocin producing cultures was modest but significant. Synergistic activity of bacteriocins and pressure against *E. coli* was also demonstrated in a meat model system (Garriga et al., 2002). Combination of the lactoperoxidase system and 600 MPa resulted in a reduction of cell counts of *E. coli* MG1655 by 4 log (cfu/mL) while use of lactoperoxidase or pressure alone was not bactericidal (Garcia-Graells et al., 2000).

## Pressure Mediated Damage to the Cytoplasmic Membrane, pH Homeostasis, and Osmoregulation

**FIGURE 2:**
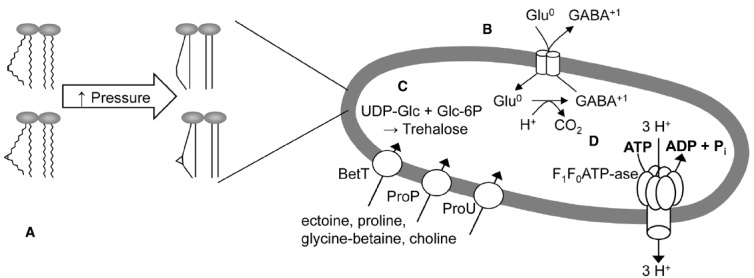
**Pressure effects on the cytoplasmic membrane and membrane bound proteins in *E. coli*. (A)** High pressure decreases lateral motion and induces phase transition in the phospholipid bilayers of *E. coli*, and promotes gelation of the membrane lipids (Pagan and Mackey, 2000; Winter, 2002; Mañas and Mackey, 2004). Pressure resistance is influenced by membrane fluidity and fatty acid composition (Casadei et al., 2002). Exponential phase cell are more sensitive to pressure when compared to stationary phase cells (Pagan and Mackey, 2000; Casadei et al., 2002). Stationary phase cell express *cfa* encoding for cyclopropane fatty acyl phospholipid synthase (CFA). CFA converts unsaturated fatty acids to cyclopropane fatty acids, which contribute to acid resistance (Brown et al., 1997; Grogan and Cronan, 1997) and pressure resistance in *E. coli* (Charoenwong et al., 2011). **(B)** Sublethal pressure inactivates acid resistance in *E. coli*. The glutamate decarboxylase system for acid resistance is more resistant to pressure than other acid resistance mechanisms, and glutamic acid decarboxylation improved the survival of *E. coli* during post-pressure acid challenge (Kilimann et al., 2005). **(C)** The accumulation of compatible solutes including glycine-betaine, choline and sucrose, and the synthesis of trehalose protects against pressure-induced cell death (Van Opstal et al., 2003; Molina-Höppner et al., 2004; Charoenwong et al., 2011); BetT, ProP, and ProU are the major transporters for compatible solutes in *E. coli*. Mutants that are defective in trehalose synthesis exhibit a reduced resistance to pressure (Charoenwong et al., 2011). **(D)** Pressure inactivates F_0_F_1_-ATPase, which causes disruption of the acid efflux system (Wouters et al., 1998).

Bacterial membranes are among the most pressure sensitive targets in bacterial cells. An overview on pressure-mediated damage to the cytoplasmic membrane is provided in Figure 2. Pressure application induces a phase transition from the physiological, liquid-crystalline phase to the gel phase (Winter, 2002). The pressure-induced phase transition of the cytoplasmic membrane also inhibits membrane bound enzymes (Wouters et al., 1998) and dissipates the proton motive force (Molina-Gutierrez et al., 2002). The *in vivo* observation of pressure-induced membrane phase transitions was achieved in *Lactobacillus plantarum* and *Lactococcus lactis* (Molina-Gutierrez et al., 2002; Ulmer et al., 2002) but not in *E. coli*, where observations of phase transitions of the cytoplasmic membrane are confounded by the outer membrane. The rapid dissipation of the proton motive force by pressure, however, was confirmed in *E coli* by *in situ* observation of the pH-dependent GFP fluorescence (Kilimann et al., 2005). Pressure as low as 10 MPa inhibits motility and substrate transport in *E. coli* (Bartlett, 2002). Remarkably, transport enzymes that are related to pH homeostasis of *E. coli* exhibit a differential resistance to pressure. Treatment of *E. coli* with 300 MPa inactivated arginine- and glucose dependent pH homeostasis but not the glutamate decarboxylase system (Figure 2; Kilimann et al., 2005). Pressure resistance is influenced by membrane fluidity and fatty acid composition (Casadei et al., 2002). Exponential phase cell are more sensitive to pressure when compared to stationary phase cells (Pagan and Mackey, 2000; Casadei et al., 2002). Stationary phase cells of *E. coli* convert unsaturated membrane lipids to cyclopropane fatty acids (Brown et al., 1997; Grogan and Cronan, 1997). Stationary phase cells also have a higher degree of crosslinking among membrane proteins and are less prone to lateral phase transition (Mirelman and Siegel, 1979; Souzu, 1986). Disruption of the cyclopropane fatty acid synthase has a decisive influence on the pressure resistance of *E. coli* (Charoenwong et al., 2011), confirming the prominent role of membrane properties in pressure-mediated cell death.

Pressure resistance of bacterial cells is intimately linked to osmoregulation and the expression of outer membrane porins. Uptake of compatible solutes in response to osmotic up-shock generally increases bacterial resistance to pressure (Molina-Höppner et al., 2004; Smiddy et al., 2004); sucrose concentrations exceeding 30% also protect *E. coli* against pressure-mediated cell death (Van Opstal et al., 2003). *E. coli* responds to osmotic up-shock by import of ectoine, proline, glycine-betaine, and choline, and by synthesis of trehalose (Figure 2; Sleator and Hill, 2002; Charoenwong et al., 2011). Disruption of trehalose biosynthesis substantially reduced the resistance of *E. coli* to pressure (Malone et al., 2006; Charoenwong et al., 2011). It is noteworthy that piezophilic adaptation to pressure also includes the accumulation of compatible solutes (Bartlett, 2002).

The strong link between bacterial adaptation to high osmotic pressure and their resistance to pressure makes the pressure-mediated elimination of *E. coli* in foods with low water activity challenging or even impossible. However, pressure mediated membrane damage and disruption of pH homeostasis allows the elimination of *E. coli* in acidic food products, particularly fruit juices (Table 1, Garcia-Graells et al., 1998; Jordan et al., 2001). The loss of acid resistance results in a pH- and pressure dependent reduction of cell counts within a few days after pressure treatment (Garcia-Graells et al., 1998; Jordan et al., 2001). It remains unclear, however, whether the substantial difference of pressure resistance of *E. coli* in fruit juices relates only to the low pH, or also involves presence (or absence) of other food constituents that affect pressure resistance in *E. coli*. For example, the inactivation of *E. coli* in fruit juices is strongly enhanced by essential oils (Espina et al., 2012).

## Pressure-mediated Damage to Cytoplasmic Components: Ribosomes, Oxidative Stress, and Protein Folding

**FIGURE 3:**
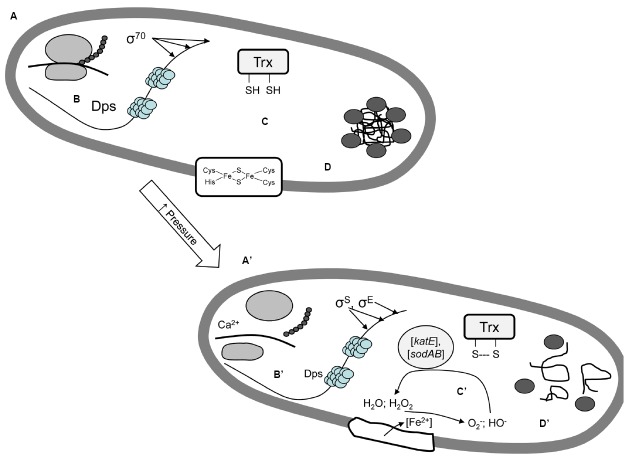
**Pressure effects on cytoplasmic proteins and nucleic acids in *E. coli*. (A,A’)** Pressure dissociates ribosomes and inhibits protein synthesis; ribosomes are stabilized by addition of divalent cations (Hauben et al., 1998; Niven et al., 1999; Gayan et al., 2013). **(B,B’)** Dps (DNA binding protein from starved cells) binds DNA as homo-dodecamer and protects *E. coli* against oxidative stress-, pressure-, and acid-induced DNA damage (Choi et al., 2000; Zhao et al., 2002; Malone et al., 2006). Deletion of the genes coding for the alternative sigma factors σ^E^ or σ^S^ increases the sensitivity of *E. coli* to pressure; indicating that the general stress response (σ^S^) and the heat shock response (σ^E^) increase pressure resistance (Robey et al., 2001; Aertsen et al., 2004, 2005; Malone et al., 2006). **(C,C’)** High pressure-induces oxidative stress in *E. coli*. Proteins that protect against peroxide and superoxide stress (thioredoxin, catalase, superoxide dismutase, and proteins that regulate their expression) also increase pressure resistance in *E. coli* (Aertsen et al., 2005; Malone et al., 2006; Charoenwong et al., 2011). The presence of iron and iron sulfur cluster proteins increases the lethality of pressure on *E. coli* (Malone et al., 2006; Yan et al., 2013), likely because free intracellular iron accumulates and catalyses the formation of reactive oxygen species. **(D,D’)** Pressure disassembles protein aggregates bodies *in vivo*, re-growth of sublethally injured cells after pressure treatment is dependent on the time required for re-assembly of protein aggregates. The presence of the locus of heat resistance which predominantly encodes genes involved in protein folding and protein turnover is generally associated with pressure resistance in *E. coli* and loss of the locus of heat resistance reduces the pressure resistance in *E. coli* AW1.7 (Garcia-Hernandez et al., 2015; Liu et al., 2015; Mercer et al., personal communication). Deletion of the inclusion body binding proteins IbpA and IbpB decreases pressure resistance (Charoenwong et al., 2011). The heat shock proteins DnaK and DnaJ contribute to assembly and segregation of protein aggregates (Aertsen et al., 2004; Govers et al., 2014), and mediate pressure resistance.

An overview on pressure-induced changes to cytoplasmic components is provided in Figure 3. *E. coli* incubated at an inhibitory but sublethal pressure of 55 MPa respond by over-expression of heat shock proteins and ribosomal proteins, suggesting that protein synthesis and protein folding are major targets for pressure-mediated cell death and injury (Welch et al., 1993).

Pressure dissociates ribosomes and inhibits protein synthesis (Niven et al., 1999, Figure 3A). Ribosomes are stabilized by addition of divalent cations (Niven et al., 1999). Pressure-induced changes to the ribosome and DNA damage are less pronounced in stationary-phase cells of *E. coli*, possibly reflecting the protective effect of the σ^S^ mediated overexpression of stress proteins preventing DNA damage (Mañas and Mackey, 2004). A direct relationship of ribosome dissociation, accumulation of compatible solutes and cellular survival was shown for heat resistance (Pleitner et al., 2012) but not for pressure resistance in *E. coli* AW1.7. The significant baro-protective effect of divalent cations on *E. coli* may partially relate to the stabilization of ribosomes (Hauben et al., 1998; Niven et al., 1999; Gayan et al., 2013).

The relationship between protein (mis)-folding, protein turnover and pressure resistance was also initially suggested by Welch et al. (1993). Resistance of *E. coli* to lethal pressure also relates to the expression of heat shock proteins (Aertsen et al., 2004; Figure 3D). Although results obtained in different studies are not always consistent, disruption of genes coding for the cold shock protein CspA, the heat shock proteins DnaK and DnaJ, and the chaperones IbpAB decrease resistance of *E. coli* to pressure (Malone et al., 2006; Charoenwong et al., 2011; Govers et al., 2014). Direct evidence for the relationship between protein (mis)-folding and pressure resistance was provided by Govers et al. (2014). Exposure of *E. coli* to 300 MPa dissociated GFP-labeled aggregates of misfolded proteins. Remarkably, the lag time of individual cells after pressure treatment was correlated to the time required for the re-assembly of protein aggregates (Govers et al., 2014).

Pressure treatment of *E. coli* in buffer systems inflicts oxidative stress (Figure 3C). Aertsen et al. (2005) directly quantified oxidative stress using cytoplasmic alkaline phosphatase as a probe. Pressure application strongly increased the oxidation of cytoplasmic disulfide bonds. The disruption of genes related to protection against oxidative stress (catalase and superoxide dismutase) decreased resistance to pressure. Malone et al. (2006) confirmed that genes providing protection against oxidative stress (DbpS, thioredoxin) also confer resistance to pressure. Remarkably, the deletion of genes coding for assembly of iron-sulfur clusters increased the resistance of *E. coli* to pressure (Malone et al., 2006), and intracellular free iron accelerates pressure-mediated cell death (Yan et al., 2013). Taken together, these studies indicate that pressure denatures proteins containing iron-sulfur clusters, resulting in the accumulation of iron in the cytoplasm. Iron catalyzes the formation of reactive oxygen species, causing oxidative stress. Consequently, proteins that detoxify reactive oxygen species, and proteins that are involved in the cytoplasmic redox-homeostasis also increase resistance to pressure (Aertsen et al., 2005; Malone et al., 2006; Charoenwong et al., 2011).

Many of the proteins involved in pressure resistance of *E. coli* are stress proteins and their expression is governed by stress-responsive alternative sigma factors, including σ^E^ or σ^S^. Deletion of *rpoE* coding for σ^E^ decreased the stress resistance of *E. coli* (Malone et al., 2006) but proteins of the σ^E^ regulon that are responsible for this effect remain to be identified. The σ^S^ regulon plays a central role in the general stress response of *E. coli*, many of the proteins that contribute to baroresistance are up-regulated by σ^S^ (Landini et al., 2014). Examples include osmoresponsive outer membrane proteins, cyclopropane fatty acid synthase, Dps, catalase, and superoxide dismutase (Landini et al., 2014, Figures 2 and 3). Consequently, deletion of *rpoS* strongly increases the sensitivity of *E. coli* to pressure (Aertsen et al., 2005; Malone et al., 2006; Charoenwong et al., 2011). Loss of the anti-σ^S^ regulator RssB increased resistance of *E. coli* to 300 MPa (Vanlint et al., 2013b). The variation of pressure resistance between different strains of *E. coli* relates to σ^S^ sequence diversity (Robey et al., 2001) and exposure of *E. coli* to sublethal pressure selects for σ^S^ activity (Vanlint et al., 2013a).

Despite the involvement of the stress-responsive σ^E^ and σ^S^ in pressure resistance of *E. coli*, a general correlation of pressure resistance to the resistance to other stressors has not been observed. Detailed information for more than 100 strains is available on the correlation of heat- and pressure resistance in *E. coli* (Liu et al., 2015). Pressure-resistant mutant strains of *E. coli* also exhibit elevated heat resistance (Hauben et al., 1997) and extremely heat resistant strains of *E. coli* are also resistant to pressure (Garcia-Hernandez et al., 2015; Liu et al., 2015). Extreme heat resistance in *E. coli* is conferred by the locus of heat resistance, a 14 kb genomic island containing 16 predicted open reading frames encoding putative heat shock proteins and proteases (Mercer et al., personal communication). Loss of the locus of heat resistance is accompanied by a substantial increase of the sensitivity to thermal inactivation (Pleitner et al., 2012) but causes only a modest increase of the sensitivity to pressure (Liu et al., 2015). Moreover, pressure resistance is also observed in strains that do not harbor the locus of heat resistance (Liu et al., 2015; Mercer et al., personal communication), demonstrating that multiple routes of acquiring pressure resistance exist in *E. coli*.

The loss of genetic material that appears unrelated to the stress response in *E. coli* increases its resistance to pressure (Malone et al., 2006; Vanlint et al., 2013b). The loss of genetic material is readily achieved by laboratory selection for resistance to pressure or membrane perturbators (Vanlint et al., 2012; Pleitner et al., 2012). The loss of genetic material rather than specific mutations may account for the ease of selection of pressure-resistance in *E. coli* (Hauben et al., 1997; Vanlint et al., 2012) as well as the large variation of pressure resistance in the species (Table 1, Liu et al., 2015).

Pressure effects on cytoplasmic proteins account for some of the synergistic or antagonistic interaction of pressure with food constituents. The baroprotecive effect of Ca^2+^ and Mg^2+^ may relate to the resistance of *E. coli* in milk and meat (Table 1). The concentration of free cytoplasmic iron correlates to pressure-induced cell death (Yan et al., 2013), however, the iron-rich meat matrix supports a high pressure resistance of *E. coli* (Table 1). The survival of pressure treated *E. coli* is significantly improved when incubated anaerobically compared to aerobic incubation (Aertsen et al., 2005) and pressure-treated foods are generally packaged without inclusion of air. A systematic screening of natural antimicrobial inhibitors revealed that only thiol-reactive inhibitory compounds exhibit synergistic antimicrobial activity with pressure (Feyaerts et al., 2015). This finding directly relates the mode of action of antimicrobial compounds to the “oxidative suicide” mechanism of pressure-mediated cell death (Figure 3; Feyaerts et al., 2015); however, this synergistic activity remains to be documented in food.

## Concluding Remarks

Research in the past two decades has identified multiple pressure-sensitive targets in *E. coli* that contribute to sublethal injury and cell death, including the composition and barrier properties of the outer and cytoplasmic membranes, ribosome assembly and functionality, protein folding, and oxidative stress caused by metabolic imbalance and/or the release of iron from denatured proteins (Figures 1,2, and 3). It remains unclear whether these targets are simultaneously or sequentially affected during high pressure treatment; however, survival during pressure treatment and post-pressure survival under adverse conditions are based on different mechanisms. Corresponding to the multiple pressure-sensitive targets in *E. coli*, pressure resistance is apparently a multi-factorial phenotype. The high frequency of *E. coli* strains with extreme pressure resistance (Liu et al., 2015) as well as the reproducible occurrence of pressure resistant mutant strains (Vanlint et al., 2012) indicates that several alternative routes to pressure resistance exist in *E. coli*. Some of these apparently include the mere loss of a few genes with no direct relation to membrane stress or the stress response (Vanlint et al., 2013b).

Owing to the multi-faceted pressure resistance of *E. coli* and the multiple factors influencing post-pressure survival in foods, the elimination of *E. coli* with high pressure as sole preservation step remains challenging or impossible. Current knowledge allows the safe elimination of *E. coli* by pressure treatment at a moderately elevated temperature, or by combination of pressure treatment at a pH of less than 4.5 in combination with a post-treatment incubation period to eliminate sublethally injured cells (Table 1). The improved knowledge on mechanisms of pressure-induced cell death and sublethal injury in *E. coli* as well as an improved understanding of the physiological and genetic determinants of pressure resistance in *E. coli* may allow the development of additional hurdle technologies to achieve food preservation with high pressure technology.

### Conflict of Interest Statement

The authors declare that the research was conducted in the absence of any commercial or financial relationships that could be construed as a potential conflict of interest.
